# The burden of peptic ulcer disease in China, 1990–2021: update from the GBD 2021 study

**DOI:** 10.1080/07853890.2026.2634612

**Published:** 2026-02-28

**Authors:** Bei Chen Guo, Guang Ming Niu, Yu Han Li

**Affiliations:** a Department of Gastroenterology and Hepatology, The University of Hong Kong-Shenzhen Hospital, Shenzhen, China; b Department of Hematology, Peking University First Hospital, Peking University, Beijing, China

**Keywords:** Peptic ulcer disease, burden of disease, China, GBD 2021, epidemiology

## Abstract

**Background:**

Peptic ulcer disease (PUD) remains a significant digestive health concern in China, influenced by factors such as *Helicobacter pylori* infection, non-steroidal anti-inflammatory drugs use and demographic changes. A comprehensive assessment of its long-term burden is crucial for public health planning.

**Methods:**

Using data from the Global Burden of Disease (GBD) 2021 study, we analysed incidence, mortality and disability-adjusted life years (DALYs) of PUD in China from 1990 to 2021. Estimated annual percentage changes (EAPCs) were calculated to assess temporal trends. Decomposition analysis was applied to quantify the contributions of population ageing, population growth and epidemiological changes to changes in absolute burden. In addition, a Bayesian age–period–cohort model was used to project trends from 2022 to 2035.

**Results:**

From 1990 to 2021, the age-standardized incidence rate of PUD in China decreased by 54.9% (EAPC = −2.85), the age-standardized mortality rate (ASMR) decreased by 73.7% (EAPC = −4.17) and the age-standardized DALY rate decreased by 77.1% (EAPC = −4.68). Despite these declines, the absolute burden remained substantial among adults aged ≥55 years. Males consistently experienced a higher burden than females. Smoking was identified as the only risk factor quantified in the GBD attribution framework for PUD. Decomposition analysis indicated that epidemiological changes accounted for the largest relative share of the observed decline, whereas population ageing partially offset these reductions. Model-based projections suggest that age-standardized rates may continue to decline through 2035, assuming recent trends persist.

**Conclusions:**

Although the age-standardized burden of PUD in China has declined markedly over the past three decades, the absolute burden remains considerable, particularly among older adults and males. Continued reductions in PUD burden may benefit from sustained tobacco control and targeted prevention strategies for high-risk populations, while accounting for the challenges posed by population ageing.

## Introduction

Peptic ulcer disease (PUD) is a prevalent disorder within the digestive system [1]. It is characterized by ulcers in the gastrointestinal mucosa, typically in the stomach or duodenum, which can extend into the muscularis mucosae [2]. The pathogenesis of PUD involves an imbalance between gastric acid–pepsin activity and mucosal defence mechanisms [3]. Infection with *Helicobacter pylori* is a significant aetiological factor contributing to the development of PUD [4,5]. In addition, non-steroidal anti-inflammatory drug (NSAID) use, smoking, alcohol consumption and psychological stress have been recognized as important risk factors for PUD [6].

Although PUD is more commonly diagnosed in adults, it also occurs in children and adolescents [7]. Paediatric PUD is relatively uncommon, but it may be clinically relevant because complications such as upper gastrointestinal bleeding and perforation can be severe [8]. A recent systematic review of perforated peptic ulcers in children summarized 12 studies (239 patients) and highlighted a male predominance and the frequent duodenal location of perforations, highlighting the clinical relevance of severe paediatric presentations [9].

PUD incurs a substantial disease burden worldwide [10]. In recent years, although the incidence and mortality of PUD may have decreased in certain regions, attributable to the extensive implementation of *H. pylori* eradication therapy and the wide use of proton pump inhibitors, it remains a significant cause of hospitalization, complications (such as bleeding, perforation and obstruction) and even mortality [11–13]. Understanding long-term epidemiological trends of PUD is essential for informing public health planning and clinical decision-making. The Global Burden of Disease (GBD) study offers comprehensive epidemiological data on diseases, serving as an invaluable resource for evaluating the global and regional disease burden [14,15]. The GBD 2021 study provides a standardized data framework that enables the assessment of long-term trends in PUD.

As the most populous country in the world, changes in the disease burden of China have important implications for global health. The epidemiological characteristics of PUD in China can be affected by multiple factors [16–18]. These include its distinctive dietary habits, the accessibility of medical services, the prevalence of *H. pylori* infection and the transformation of the population structure. Notwithstanding, comprehensive and long-term epidemiological research focusing on the disease burden of peptic ulcer in China remains relatively scarce.

In this study, we analysed data from the GBD 2021 to characterize long-term trends in the incidence, mortality and burden of PUD in China from 1990 to 2021. Analyses were stratified by sex and age to explore demographic disparities. We further applied decomposition analysis to quantify the contributions of population ageing, population growth and epidemiological changes to variations in the absolute burden of PUD. In addition, a Bayesian age–period–cohort (BAPC) model was used to project future trends in PUD burden from 2022 to 2035.

## Methods

### Data retrieval and preparation

All data were obtained from the GBD 2021 Results Tool (http://ghdx.healthdata.org/gbd-results-tool), which integrates epidemiological information from 204 countries and territories across 21 GBD regions. We extracted annual estimates for China from 1990 to 2021 for PUD, including incidence, deaths and disability-adjusted life years (DALYs), reported as both absolute counts and age-standardized rates with corresponding 95% uncertainty intervals (UIs). Data were stratified by sex and age groups as provided by the GBD framework. Within the GBD cause hierarchy, PUD comprises gastric ulcer, duodenal ulcer, peptic ulcer of unspecified site and gastrojejunal ulcer, as defined by the corresponding ICD-9 and ICD-10 codes mapped in the GBD cause list. We did not apply any additional inclusion or exclusion criteria beyond the standard GBD case definition and modelling procedures. Risk-attributable estimates for PUD were obtained for smoking only, as smoking is the only risk factor with available PUD-attributable deaths and DALYs in the GBD Results framework for the GBD 2021 release. No post hoc filtering or subgroup exclusion was performed. Extracted data were cleaned and formatted for subsequent trend analysis, decomposition analysis and BAPC modelling.

### Outcomes

The primary outcomes were age-standardized incidence rate (ASIR), age-standardized mortality rate (ASMR) and age-standardized DALY rate (ASDALYR) (per 100,000 population) of PUD in China from 1990 to 2021. These age-standardized rates were selected as primary outcomes because they facilitate comparisons across time by accounting for changes in the population age structure.

Secondary outcomes included the corresponding absolute numbers (incident cases, deaths and DALYs), age- and sex-stratified estimates, percent change from 1990 to 2021, estimated annual percentage changes (EAPCs), decomposition components, risk-attributable burden estimates available in the GBD framework and BAPC-based projections for 2022–2035. All outcomes were obtained as GBD modelled estimates rather than individual-level observations. Therefore, results are subject to limitations inherent to secondary model-based data.

### EAPC estimation

The EAPCs of the age-standardized rates for PUD were calculated to assess trends between 1990 and 2021 [19]. We fitted a regression line to the natural logarithm of the age-standardized rates, that is, y = α + βx + ε, where y = ln (rates) and x = calendar year and ε = error term. The calculation of the EAPCs in age-standardized rates and their 95% confidence interval (CI) was performed as 100* (exp(β)–1). An increasing trend was inferred when the 95% CI of the EAPC was entirely above zero, whereas a decreasing trend was inferred when the 95% CI was entirely below zero.

### Decomposition analysis of changes in PUD burden

To quantify the drivers of changes in the absolute burden of PUD in China between 1990 and 2021, we conducted a decomposition analysis partitioning the net change in counts into three components: population growth, population ageing and epidemiological change [20,21].

We applied the Das Gupta stepwise replacement method, which has been widely used in GBD analyses. Briefly, the observed difference in absolute counts between 1990 and 2021 was decomposed by sequentially replacing population size, age structure and age-specific rates from the baseline year to the comparison year, while holding the remaining components constant. Epidemiological change, therefore, reflects variations in age-specific rates after accounting for demographic shifts. For each outcome (incidence, deaths and DALYs), the absolute contribution of each component was calculated as the number of cases, deaths, or DALYs attributable to that component. When the overall net change was negative (i.e. a net decline in absolute burden), components that increased counts – such as population ageing or population growth – appear as negative percentage contributions because they offset part of the decline. Percentage values may exceed ±100% when different components act in opposite directions, partially cancelling each other out. Decomposition results should be interpreted as a descriptive partitioning of observed changes rather than as evidence of causal effects.

### BAPC model for projection

To project future trends in PUD burden in China from 2022 to 2035, we applied a BAPC model implemented using integrated nested Laplace approximation (INLA) [22].

The BAPC framework decomposes temporal variation in disease rates into age, period and cohort components, while addressing the identifiability constraints of classical APC models through Bayesian smoothing. Adjacent age groups, calendar periods and birth cohorts were assumed to share similar underlying risk structures. To stabilize estimates and reduce overfitting, second-order random walk priors were specified for age, period and cohort effects, consistent with established BAPC applications in disease burden forecasting.

Observed counts (incidence, deaths, or DALYs) were assumed to follow a Poisson distribution, with expected counts modelled as a log-linear function of age, period and cohort effects, incorporating population size as an offset. INLA provides fast and accurate approximations of posterior marginal distributions without reliance on Markov chain Monte Carlo sampling and is well-suited for large-scale population data.

Posterior means and 95% credible intervals were derived directly from the marginal posterior distributions. Because INLA does not rely on iterative Markov chain Monte Carlo chains, traditional convergence diagnostics are not required; model adequacy was assessed through internal consistency checks and comparison of fitted and observed values during the historical period.

The BAPC model was fitted using GBD 2021 data from 1990 to 2021. Projections were generated by extrapolating period and cohort effects beyond 2021 under the assumption that recent temporal patterns would persist, while maintaining the same prior smoothness structure. Age-specific population projections from the GBD were incorporated as offsets.

Uncertainty in projected estimates reflects both parameter uncertainty from the Bayesian model and uncertainty in the underlying GBD inputs. All projected results are therefore reported with 95% uncertainty intervals.

Importantly, BAPC projections represent scenario-based forecasts conditional on historical trends and modelling assumptions, rather than deterministic predictions. Consequently, future changes in demographic structure, healthcare access, clinical practice, or population-level risk factors may lead to deviations from the projected trajectories.

### Statistical considerations and software

All analyses were based on GBD 2021 estimates, which constituted the most recent complete and validated GBD release available at the time of data extraction and study conduct. All statistical analyses and data visualizations were performed using R (version 4.4.2) and JD_GBDR (version 2.37, Jingding Medical Technology Co., Ltd.). Because this study used aggregate GBD estimates (counts and rates with UIs), rather than individual-level continuous variables, normality testing was not applicable. We therefore present descriptive results as point estimates with 95% UIs and EAPCs with 95% CIs. All rates are expressed per 100,000 population unless otherwise specified.

## Results

### Trends in the burden of PUD in China from 1990 to 2021

**Figure 1.**
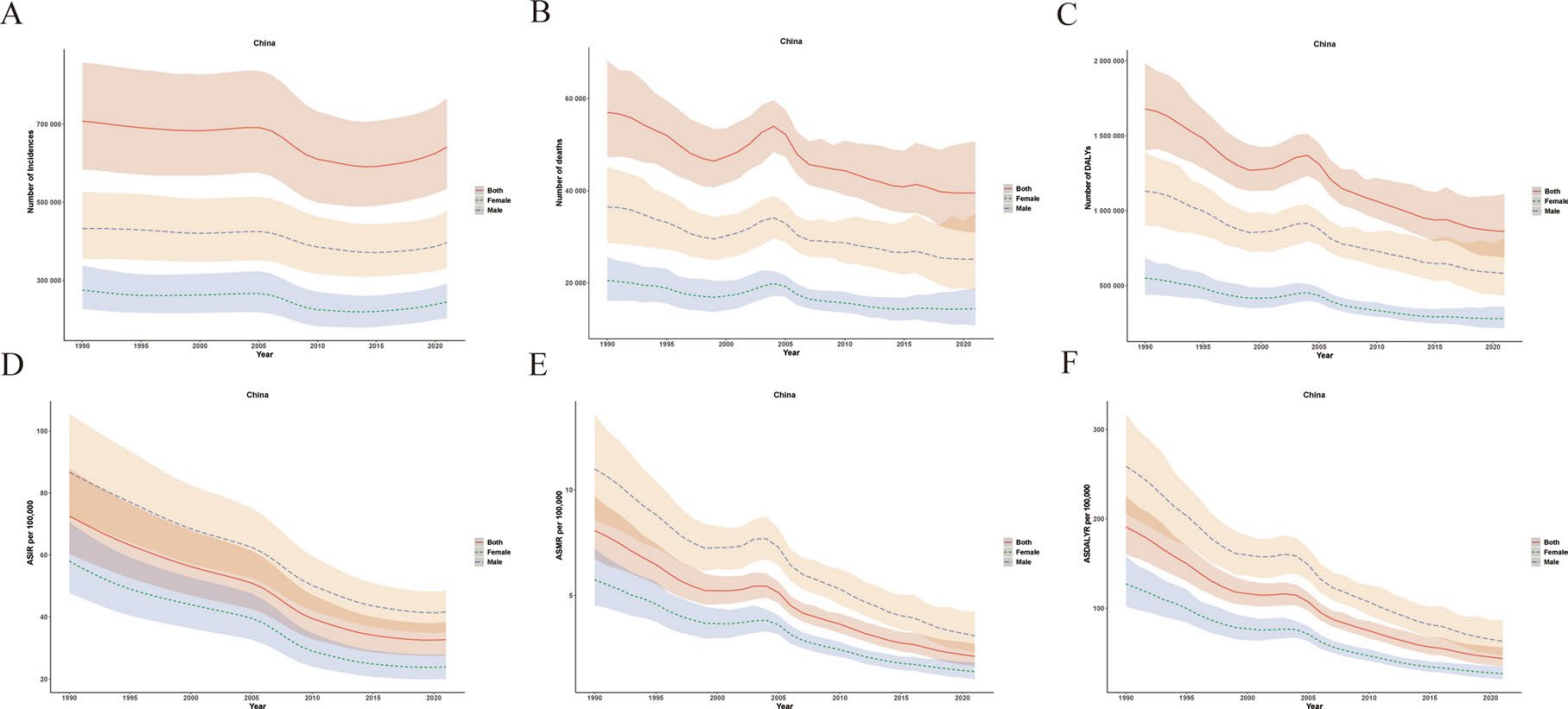
Numbers and Age-specific rates of PUD in China from 1990 to 2021 for males, females and both sexes. (A) Incidence number. (B) Mortality number. (C) DALYs number. (D) Age-specific incidence rate. (E) Age-specific mortality rate. (F) Age-specific DALYs rate. *Note:* Shaded areas/error bars represent 95% UIs for GBD estimates.

According to an analysis of the GBD database, the number of incidence cases of PUD in China across all age groups in 1990 was 707,561 (95% UI 583,387, 858,039), while in 2021, it decreased to 641,108 cases (95% UI 533,979, 766,093), representing a 9.39% decline (Table 1 and Figure 1(A)). The ASIR decreased from 72.58 (95% UI 60.35, 87.82) in 1990 to 32.71 (95% UI 27.58, 38.37) in 2021, a decrease of 54.9%, showing a decreasing trend year by year, with an EAPC of −2.85 (95% CI −3.00, −2.70) (Table 1 and Figure 1(D)). The total number of deaths from PUD in China decreased from 57,007 cases (95% UI 47,279, 68,220) in 1990 to 39,499 cases (95% UI 30,912, 50,678) in 2021, representing a decrease of 30.7% (Table 1 and Figure 1(B)). The ASMR decreased from 8.07 (95% UI 6.74, 9.71) in 1990 to 2.12 (95% UI 1.66, 2.70) in 2021, a decrease of 73.7%, with an EAPC of −4.17 (95% CI −4.42, −3.93) (Table 1 and Figure 1(E)). The burden of mortality showed a significant downward trend from 1990 to 2021, but there was a slight fluctuation between 2000 and 2005 (Figure 1(B,E)). The DALYs burden of PUD in China were similar to the mortality burden. In 1990, the total DALYs were 1,679,900 (95% UI 1,403,300, 1,983,700). By 2021, the total DALYs had decreased to 860,700 (95% UI 685,700, 1,109,700), representing a 48.8% decrease (Table 1 and Figure 1(C)). The ASDALYR decreased significantly from 190.88 (95% UI 159.92, 225.55) in 1990 to 43.79 (95% UI 35.04, 56.06) in 2021, a decrease of 77.1%, with an EAPC of −4.68 (95% CI −4.87, −4.49) (Table 1 and Figure 1(F)).

**Table 1.** Numbers, age-standardized incidence, mortality and disability-adjusted life years (DALYs) rates, PC and EAPC of PUD for China in 1990 and 2021.

	1990	2021	PC	EAPC (95% CI)
Incidence cases (95% UI)	707,561 (583,387, 858,039)	641,108 (533,979, 766,093)	−9.39 (−14.14, −3.41)	
ASIR (95% UI)	72.58 (60.35,87.82)	32.71 (27.58,38.37)	−54.93 (−57.25,−52.76)	−2.85 (−3.00,−2.70)
Death cases (95% UI)	57,007 (47,279, 68,220)	39,499 (30,912, 50,678)	−30.71 (−49.47,−1.92)	
ASMR (95% UI)	8.07 (6.74,9.71)	2.12 (1.66,2.70)	−73.73 (−80.72,−62.98)	4.17 (−4.42,−3.93)
DALYs (95% UI)	1,679,922 (1,403,269,1,983,729)	860,684 (685,715,1,109,681)	−48.77 (−62.32,−26.92)	
ASDALYR (95% UI)	190.88 (159.92,225.55)	43.79 (35.04,56.06)	−77.06 (−82.96,−67.53)	−4.68 (−4.87,−4.49)

*Note:* EAPC: estimated annual percentage change; PC: percentage change; ASIR: age-standardized incidence rates; ASMR: age-standardized mortality rates; DALYs: disability-adjusted life years; ASDALYR: age-standardized disability-adjusted life years rates; UI: uncertainty interval; CI: confidence interval. Note: Data are presented as estimates (95% UI) unless otherwise stated. EAPCs is presented as estimates (95% CI). PC indicates the percent change between 1990 and 2021..

### The burden of PUD in China by gender and age

The incidence cases of both males and females with PUD have shown a downward trend, with the males consistently remaining higher than that of females (Figure 1(A)). The ASIR of males has decreased from 86.68 (95% UI 72.23, 105.38) in 1990 to 41.64 (95% UI 35.23, 48) in 2021(Figure 1(D) and Supplementary Table 1). The ASIR of females decreased from 58.03 (95% UI 47.79, 70.65) to 23.85 (95% UI 20.03, 28.03) (Figure 1(D) and Supplementary Table 1). The deaths and DALYs burden of males with PUD are comparable to that of females, exhibiting a downward trend in general (Figure 1(B,C,E,F)). From 1990 to 2021, the ASMR for males decreased from 10.99 (95% UI 8.58, 13.57) to 3.10 (95% UI 2.29, 4.24) (Supplementary Table 1). The ASMR for females decreased from 5.75 (95% UI 4.54, 7.22) to 1.40 (95% UI 1.04, 1.82), indicating a 75% reduction (Supplementary Table 1). The ASDALYR for males decreased from 258.65 (95% UI 205.95, 316.94) in 1990 to 63.02 (95% UI 47.33, 86.55) in 2021, indicating a decline of 75.6%. Similarly, the ASDALYR for females decreased from 127.30 (95% UI 102.11, 158.01) in 1990 to 26.87 (95% UI 20.70, 34.58) in 2021, showing a decrease of 78.9% (Supplementary Table 1).

**Figure 2.**
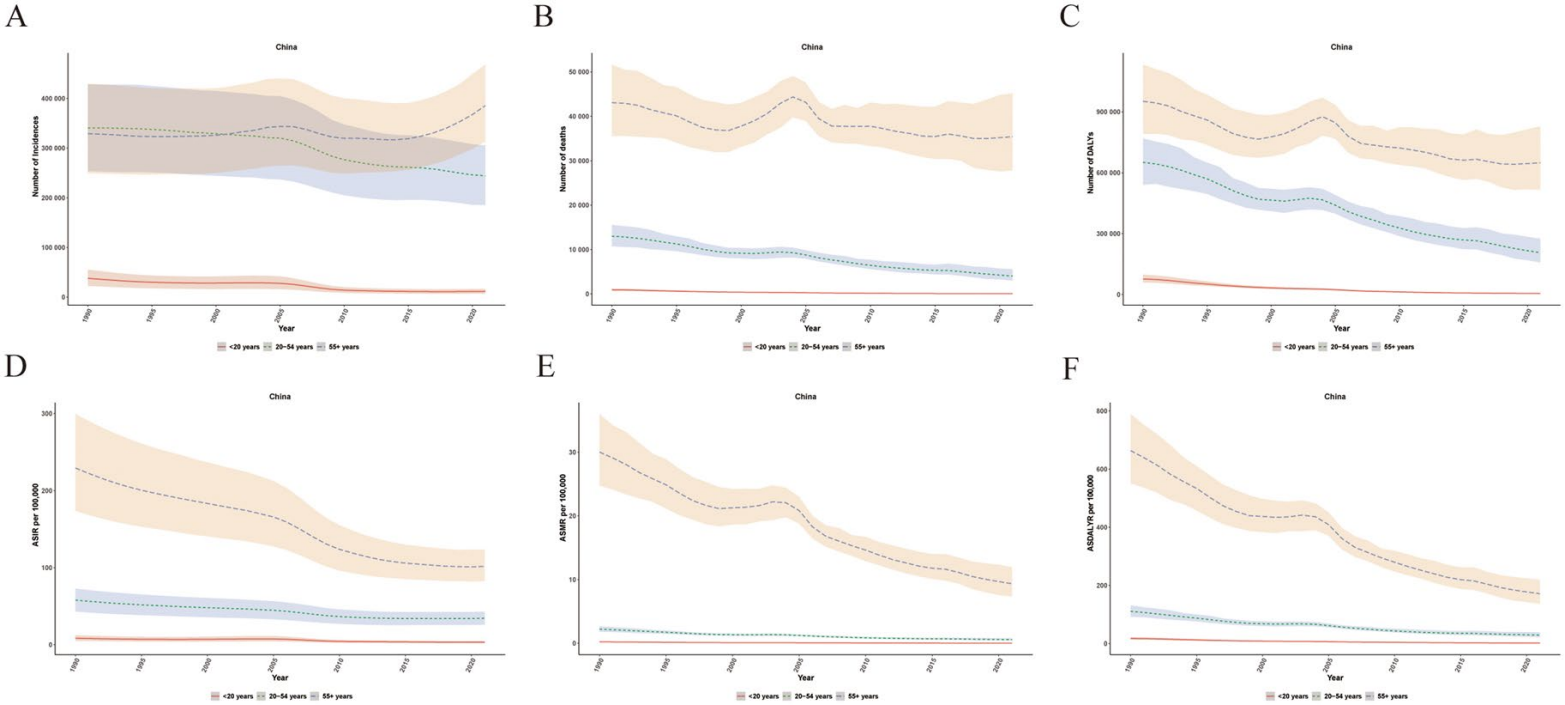
The burden of peptic ulcer disease in China from 1990 to 2021, stratified by age groups. (A) Number of incidence cases. (B) Number of deaths. (C) Number of disability-adjusted life years (DALYs). (D) Age-standardized incidence rate (ASIR) per 100,000 population. (E) Age-standardized mortality rate (ASMR) per 100,000 population. (F) Age-standardized DALY rate (ASDALYR) per 100,000 population. *Note:* Shaded areas/error bars represent 95% UIs for GBD estimates.

This study analysed the age-stratified trends of PUD in China from 1990 to 2021 using the GBD database. The number of incidence cases among those under 20 years old decreased significantly from 37,800 (95% UI 22,638, 55,329) in 1990 to 11,423 (95% UI 6,992, 16,537) in 2021, a decline of 69.8%(Figure 2(A) and Supplementary Table 2). The ASIR for those under 20 years old decreased from 8.49 (95% UI 5.09, 12.43) in 1990, steadily decreasing to 3.37 (95% UI 4.87, 2.06) in 2019 and then increased slightly to 3.42 (95% UI 4.95, 2.09) in 2021(Figure 2(D) and Supplementary Table 2). Notably, the ASIR accelerated its decline after 2006 (Figure 2(D)). Among 20- to 54-year-olds, the number of incidence cases decreased from 340,642 (95% UI 253,604, 428,069) in 1990 to 244,180 (95% UI 185,466, 305,603) in 2021, a 28% decrease (Figure 2(A) and Supplementary Table 2). From 1990 to 2015, the ASIR for 20- to 54-year-olds steadily decreased from 57.94 (95% UI 72.81, 43.14) to 34.10 (95% UI 42.52, 25.54) (Figure 2(D) and Supplementary Table 2). Since then, it has remained relatively stable (Figure 2(D)). From 2006 to 2010, the decrease was the most significant (Figure 2(D)). The number of incidence cases over 55 years old increased from 329,118 (95% UI 249,073, 429,747) in 1990 to 385,505 (95% UI 313,243, 468,258) in 2021, which is an increase of 17.1% and has consistently maintained the highest incidence rate, which was 229.32 (95% UI 299.44, 173.55) in 1990 (Figure 2(A) and Supplementary Table 2). The trends in the burden of deaths and DALYs were similar to the incidence burden (Figure 2(B,C,E,F)). There is an overall downward trend, with the highest burden in the 55+ age group (Figure 2(B,C,E,F)).

### Variation in PUD burden in two sexes and five-year age groups in China in 2021

**Figure 3.**
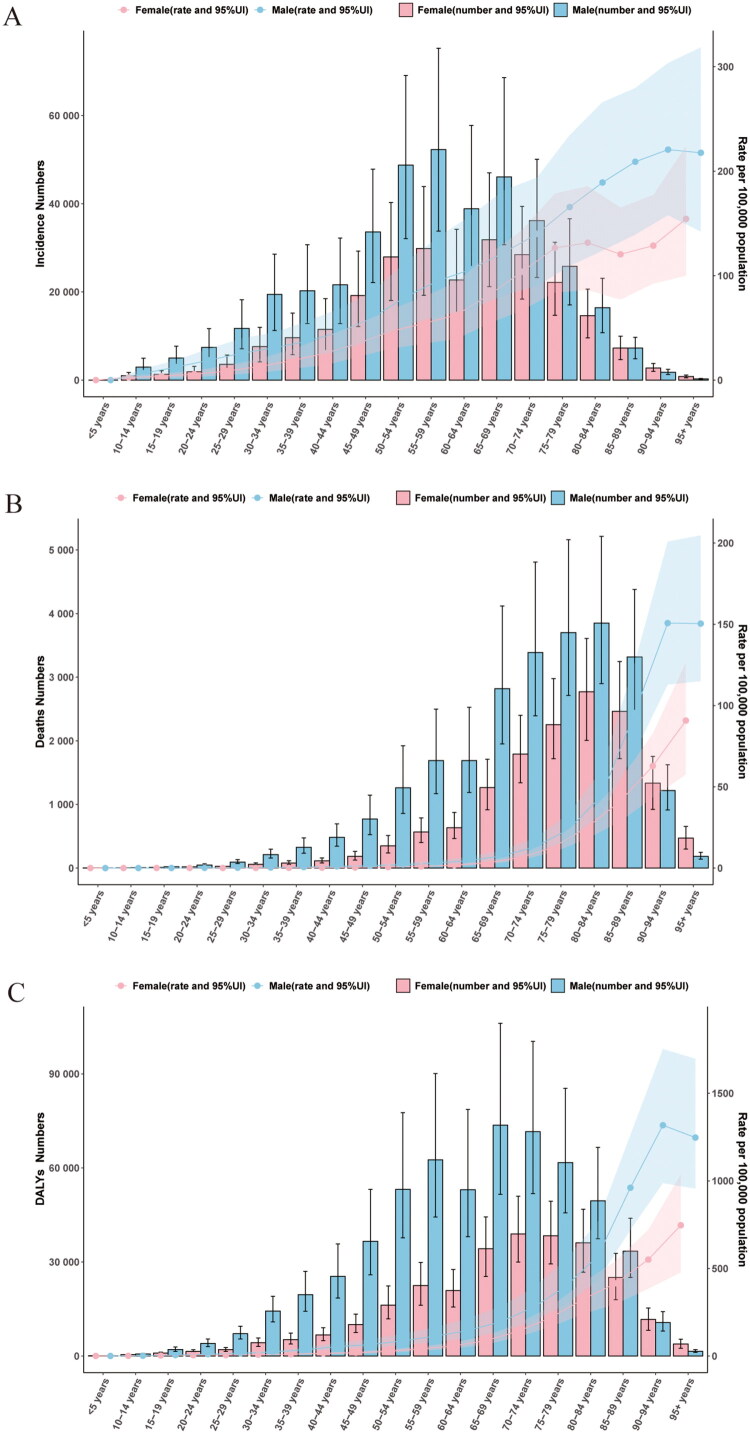
Age patterns by sex of the total number and age-specific incidence rates (A), age-specific mortality rates (B) and age-specific DALYs rates (C) due to PUD for China in 2021. *Note:* Shaded areas/error bars represent 95% UIs for GBD estimates.

In 2021, the number of incidences, deaths and DALYs for most age groups of men was higher than for women, except for those aged 90–94 and 95+(Figure 3(A–C)). The ASIR, ASMR and ASDALYR for men in all age groups were higher than for women and increased with age (Figure 3(A–C)). The age group with the highest number of incidences for men was 55–59, and for women, it was 65–69 (Figure 3(A)). The age group with the highest ASIR, ASMR and ASDALYR for men was 90–94, and for women, it was 95+(Figure 3(A–C)).

### PUD risk factors in different age groups and years in China in 2021

**Figure 4.**
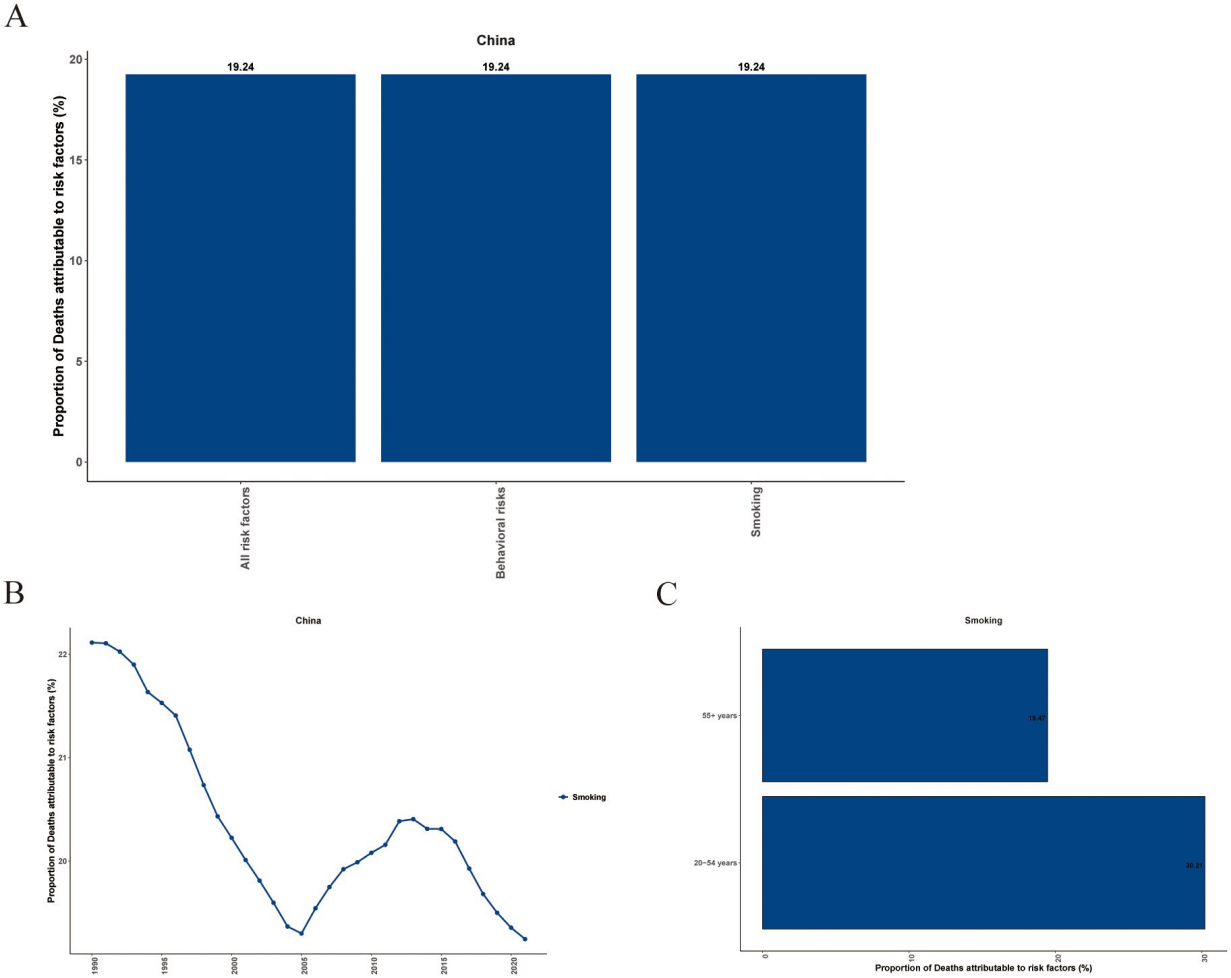
The proportion of PUD deaths attributable to smoking in China, 2021. (A) The proportion of PUD deaths attributable to smoking. (B) The temporal trend of the proportion of PUD deaths attributable to smoking from 1990 to 2021. (C) The proportion of PUD deaths attributable to smoking stratified by age group.

Regarding the risk factors for death in Chinese patients with Peptic ulcers, our downloaded data indicates one risk factor: smoking (Figure 4(A)). The risk is higher in the middle-aged group than in the elderly group in 2021(Figure 4(C)). We found that, before 2006, the risk decreased over time (Figure 4(B)). After 2006, the risk first increased and then decreased (Figure 4(B)). Overall, the risk declined. The risk factors for DALYs are similar to those for death (Supplementary Figure 1).

### Decomposition analysis of PUD in China

Decomposition analysis was conducted to partition the change in the absolute burden of PUD from 1990 to 2021 into components attributable to population growth, population ageing and epidemiological change. Overall, DALYs, deaths and incident case counts declined during this period. Under the decomposition framework, the net decreases in DALYs, deaths and incidence were largely aligned with epidemiological change, whereas population ageing and population growth exerted upward pressure and therefore offset part of the overall decline.

**Figure 5.**
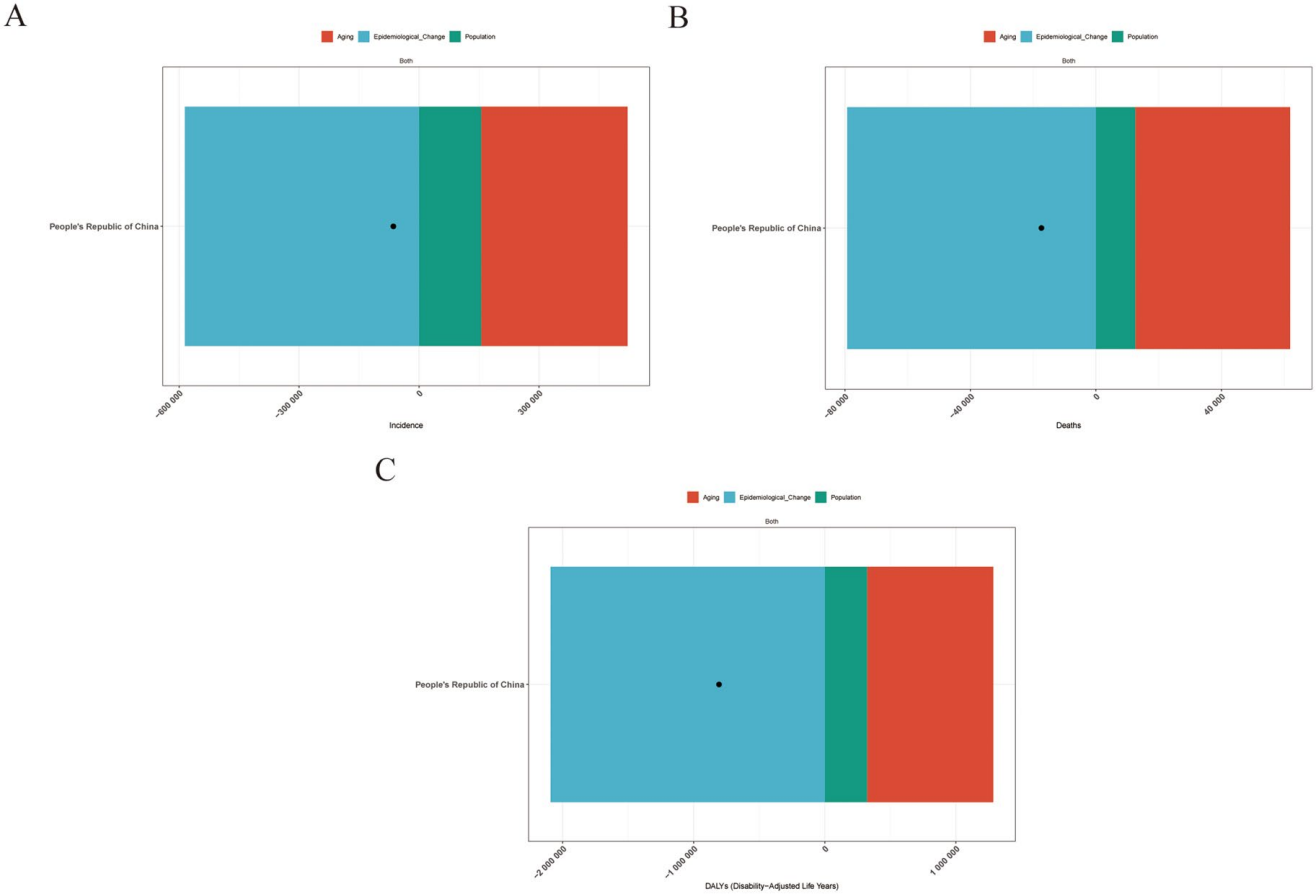
Results of the decomposition analysis on the changes in peptic ulcer disease burden in China between 1990 and 2021. (A) Contribution of factors to the change in the number of incidence cases. (B) Contribution of factors to the change in the number of deaths. (C) Contribution of factors to the change in disability-adjusted life years (DALYs). *Note:* decomposition results should be interpreted as attribution of change components rather than causal effects.

For DALYs, epidemiological change corresponded to 259.25% of the net decrease, while population ageing and population growth offset the decline (−119.21% and −40.04%, respectively) (Figure 5(C) and Supplementary Table 3). A similar pattern was observed for deaths, with epidemiological change corresponding to 456.27% of the net decrease and demographic components counteracting the decline (−283.72% for ageing and −72.55% for population growth) (Figure 5(B) and Supplementary Table 3). For incidence, epidemiological change corresponded to 913.08% of the net decrease, while population ageing and population growth offset part of this reduction (−570.59% and −242.50%, respectively) (Figure 5(A)and Supplementary Table 3). Notably, percentage contributions may exceed ±100% when components operate in opposite directions and partially cancel each other out.

### Predictions of PUD from 2022 to 2035 in China

**Figure 6.**
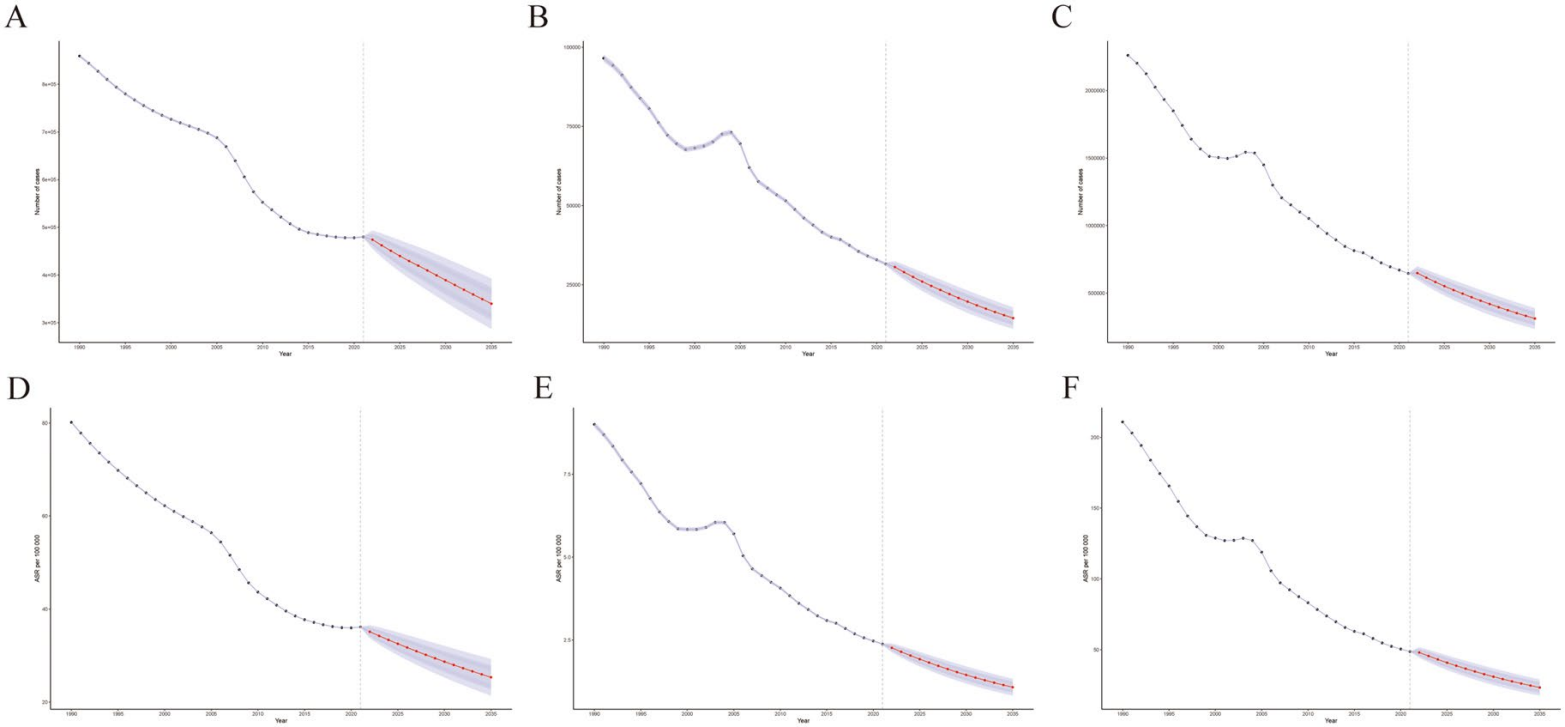
Projected trends of PUD burden in China from 2022 to 2035 using a BAPC model. (A) Projected number of incidence cases. (B) Projected number of deaths. (C) Projected number of disability-adjusted life years (DALYs). (D) Projected age-standardized incidence rate (ASIR) per 100,000 population. (E) Projected age-standardized mortality rate (ASMR) per 100,000 population. (F) Projected age-standardized DALY rate (ASDALYR) per 100,000 population.

We used a BAPC model to predict the number of incidence cases, deaths and DALYs for PUD, within the next 15 years and the corresponding ASIR, ASMR and ASDALYR. The results show that the incidence cases are expected to reach 339,881 by 2035, with an ASIR of 25.33 per 100,000 people (Figure 6(A,D)). Additionally, the death toll is projected to be 18,512 by 2035, with a rate of 1.08 per 100,000 people, a decrease of 49% from 2021(Figure 6(B,E)). The DALYs are estimated to reach 312,299 by 2035, with an ASDALY of 23.28 per 100,000 people, a decrease of 46.8% from 2021 (Figure 6(C,F)).

## Discussion

Previous GBD-based studies have described the global and regional burden of PUD, including analyses using GBD 2019 data (1990–2019), such as those by Zhang et al. [23]. While these studies provide valuable baseline evidence on long-term trends, our study builds upon them by using the GBD 2021 release (1990–2021) and providing additional analytical components beyond updated years. Stratified by gender, age and risk factors, our findings reveal disparities across different demographic groups. In particular, we quantified the demographic and epidemiological drivers of changes in absolute burden through decomposition analysis and produced scenario-based projections using a BAPC approach.

Our research shows that from 1990 to 2021, the ASIR, ASMR and ASDALYR of PUD in China all showed a downward trend, similar to the previous research results of Zhang et al. [23]. The declining trends in the incidence and mortality of PUD may reflect, at least in part, improvements in healthcare access and delivery in China, wider dissemination of *H. pylori* diagnosis and treatment and NSAID prescribing practices. Several China-specific contextual factors may also be relevant when interpreting the observed trends. Over the past decades, expanded access to healthcare services and broader population coverage by health insurance, together with improvements in diagnostic capacity and clinical management (e.g. increased availability of endoscopy, proton pump inhibitors and standardized management of upper gastrointestinal bleeding), may have contributed to declines in age-standardized rates [24,25]. At the same time, rapid population ageing and a rising prevalence of comorbidities and polypharmacy in older adults may increase susceptibility to ulcer-related complications and sustain healthcare demand despite declining standardized rates. Moreover, regional disparities in healthcare resources, urban–rural differences in access and health-seeking behaviour and heterogeneity in service delivery may contribute to uneven burden patterns within China, which cannot be fully captured by national-level GBD summaries [25]. When interpreting the long-term temporal trends, it should be noted that seasonal variation has been reported for gastrointestinal diseases and ulcer-related complications [26]. However, because the GBD framework provides annual rather than sub-annual estimates, such within-year seasonal patterns could not be assessed in the present analysis and are not captured in the trends reported here.

An important finding is the divergence between declining age-standardized incidence, mortality and DALY rates and the persistently substantial absolute burden among adults aged ≥55 years. Age-standardized rates reflect underlying risk after accounting for changes in population age structure, whereas absolute counts represent the total number of events and are strongly influenced by demographic shifts. In the context of China’s rapidly ageing population, this divergence implies that healthcare demand related to PUD may continue to increase among older adults, even as age-standardized rates decline.

Our age-stratified analyses also included paediatric age groups. Compared with adults, PUD in children and adolescents is characterized by greater aetiological and clinical heterogeneity. Clinical presentation in children may be less specific and complications such as bleeding or perforation, although uncommon, carry substantial morbidity. Recent paediatric evidence supports the clinical relevance of severe complications; for example, Vidović et al. [9]. summarized paediatric perforated PUD and reported that most cases occurred in males and were more often duodenal than gastric. Therefore, although the overall paediatric burden is smaller than that in older adults, paediatric cases warrant timely recognition and appropriate management. We also note that paediatric burden estimates in GBD may be more uncertain due to fewer primary data sources, and thus, paediatric trends should be interpreted cautiously.

Gender disparities were evident throughout the study period, with males exhibiting consistently higher rates of PUD than females, which is consistent with previous research findings [2,27,28]. This pattern may be related to a combination of biological and behavioural factors, including differences in *H. pylori* prevalence, lifestyle exposures and sex-related hormonal influences, although the underlying mechanisms remain incompletely understood [28–31]. Oestrogen and progesterone may confer protective effects on the gastric mucosa in women, whereas testosterone might exacerbate mucosal vulnerability in men [29]. In addition to potential biological mechanisms, sex-specific differences in behavioural risk profiles, comorbidity patterns, medication exposure, occupational and psychosocial stress and healthcare-seeking behaviour may also contribute to divergence in PUD burden between males and females. Given the aggregate and modelled nature of GBD estimates, these explanations should be regarded as contextual interpretations rather than definitive mechanisms.

Smoking is an independent risk factor for PUD [2]. It can augment gastric acid secretion, compromise the integrity of the gastric mucosal barrier and impede the *H. pylori* eradication [30,32,33]. This study characterized the temporal trends of smoking prevalence in China from 1990 to 2021 and examined its association with the burden of PUD. Even when the overall PUD burden is decreasing, an upward trend in smoking prevalence within specific periods or among certain populations may still render smoking a potential contributor to elevated PUD burden, either locally or in the future. Therefore, enhanced tobacco control efforts, especially those targeting high-risk populations, could play a supportive role in reducing the burden of PUD.

Although *H. pylori* infection and NSAID use are widely recognized as major aetiological determinants of PUD, the present study is based on GBD modelled estimates and does not include direct measures of *H. pylori* eradication coverage, antibiotic utilization, or NSAID use over time. Therefore, we were unable to perform within-dataset correlation analyses to quantify their contributions to temporal changes.

Decomposition analysis has shed light on the specific contributions of population ageing, epidemiological shifts and demographic changes to the changes in the disease burden of PUD. Population ageing appears to be an important demographic factor contributing to changes in the absolute burden of PUD [34]. Epidemiological changes may reflect, among other factors, the wider implementation of *H. pylori* eradication therapy and preventive strategies for NSAID-related gastrointestinal injury [33,35]. Demographic changes, encompassing urbanization and lifestyle transformations, may also exert an indirect influence on the disease burden of PUD. Importantly, our decomposition results represent a statistical partitioning of observed changes and should not be interpreted as causal effects.

Our findings should be interpreted in the context of broader GBD-based evidence. Previous global and regional analyses have reported overall declines in age-standardized rates of PUD over recent decades, while absolute counts may remain substantial or shift towards older age groups due to demographic ageing [36]. The pattern observed in China is broadly consistent with trends reported in East Asia and globally [37,38], reinforcing that declining age-standardized rates can coexist with a growing absolute burden in ageing populations. Within China, several factors may plausibly contribute to these dynamics, including rapid population ageing, evolving clinical practices and healthcare access and changes in major determinants of PUD (e.g. the epidemiology and management of *H. pylori* infection and patterns of medication exposure such as NSAID prescribing practices/antithrombotic agents in older adults) [39,40].

This study applied a BAPC model to project future incidence trends of PUD in China from 2022 to 2035. The APC framework is commonly used to disentangle age, period and cohort effects in disease rates. Compared with traditional APC approaches, the BAPC model allows for probabilistic forecasting with quantified uncertainty and improved handling of data sparsity. The projected trends provide a quantitative reference for informing future strategies for PUD prevention and control. A continued decline in incidence rates would be consistent with the maintenance of current prevention and control efforts, whereas a potential increase in specific age groups or cohorts may indicate areas where closer monitoring and more targeted public health responses could be considered.

Several limitations merit consideration. First, this study relied on modelled estimates from the GBD 2021 framework rather than individual-level clinical data. Although GBD provides internally consistent estimates with quantified uncertainty, results depend on the availability and quality of input data and on modelling assumptions and may be revised in future GBD iterations. Formal inferential testing of subgroup differences (e.g. by sex or age) is therefore not directly supported and comparisons were interpreted based on effect size and 95% UIs rather than conventional p-values. Second, despite standardized cause definitions within GBD, misclassification may occur due to variations in ICD coding practices, healthcare reporting and cause–ICD mapping, particularly for ulcer-related complications and gastrointestinal bleeding. Paediatric estimates may also be less stable because PUD is relatively rare and potentially underdiagnosed in younger age groups, resulting in wider uncertainty. Third, risk-factor attribution was limited by available GBD outputs for PUD. Only smoking-attributable burden could be assessed, whereas other key aetiological factors, including *H. pylori* infection, NSAID use and alcohol consumption, were not directly quantified. In addition, the use of aggregated modelled estimates precluded direct evaluation of time-varying contextual determinants, such as *H. pylori* eradication coverage, healthcare reforms, insurance expansion, or regional disparities; causal interpretations should therefore be avoided. Fourth, projections based on the BAPC model represent scenario-based forecasts conditional on historical trends and modelling assumptions. Future demographic changes, policy shifts, clinical practice changes, or behavioural trends may lead to deviations from projected trajectories. Finally, GBD provides annual rather than sub-annual estimates, which precludes assessment of seasonal variation. Seasonal patterns in ulcer-related outcomes may contribute to short-term fluctuations but are not captured in annual, population-level analyses.

Looking ahead, future research could further refine the classification of PUD, for instance, by differentiating between *H. pylori*-associated ulcers and non-*H. pylori*-associated ulcers, thereby furnishing more precise epidemiological insights. Additionally, a more nuanced analysis of regional disparities, integrating the distribution of regional medical resources and economic development levels, could provide valuable support for targeted prevention and control efforts at the local level. Moreover, future prospective cohort studies may provide additional insight into the complex interplay among multiple risk factors and their relationships with PUD incidence, which could support the development of more tailored prevention and intervention approaches.

## Conclusion

Based on GBD 2021 estimates, China experienced a substantial decline in the age-standardized burden of PUD between 1990 and 2021. Nevertheless, the absolute burden remains considerable, particularly among older adults and males. Decomposition analysis indicates that epidemiological changes represented the largest component of the overall reduction in PUD burden, whereas population ageing partially offset this decline by increasing absolute counts. Scenario-based projections using a BAPC model indicate that age-standardized rates may continue to decline through 2035 if recent trends persist. However, these projections should be interpreted cautiously, as they depend on modelling assumptions and may vary with future changes in demographic structure, health policies, healthcare access, clinical management and population-level risk exposures. Further reductions in the burden of PUD may benefit from sustained attention to high-risk populations and comprehensive tobacco control efforts.

## Supplementary Material

Supplement.docx

## Data Availability

The datasets supporting the conclusions of this study can be downloaded from the official website of the GBD database (https://vizhub.healthdata.org/gbd-results/) and are also available from the corresponding author upon reasonable request.
