# Supplementary material for: The burden of peptic ulcer disease in China, 1990–2021: update from the GBD 2021 study
Source: Ann Med. 2026 Feb 28;58(1):2634612. doi: 10.1080/07853890.2026.2634612 (PMC12951679; doi:10.1080/07853890.2026.2634612)
Supplement: Supplement.docx [file IANN_A_2634612_SM1472.docx]

**Supplementary Table 1.** The number and age-standardized rates of incidence, deaths, and DALYs for PUD in China by sex, 1990 and 2021.

|  | male1990 | female 1990 | male 2021 | female 2021 |
| --- | --- | --- | --- | --- |
| Incidence number  (95% UI) | 432354(354582,525833) | 275207 (227264,337344) | 396600.18(331537,477498) | 244507 (203155,291455) |
| ASIR  (95% UI) | 86.68(72.23,105.38) | 58.03(47.79,70.65) | 41.64(35.23,48.46) | 23.85(20.03,28.03) |
| Death number  (95% UI) | 36485(28750,45188) | 20523(16139,25528) | 25099(18409,34968) | 14400 (10746,18701) |
| ASMR  (95% UI) | 10.99(8.58,13.57) | 5.75(4.54,7.22) | 3.1(2.29,4.24) | 1.4(1.04,1.82) |
| DALY number  (95% UI) | 1129553 (898120,1388308) | 550368.92(439811,683013) | 581300(431993,814161) | 279384(214845,359427) |
| ASDALYR(95% UI) | 258.65(205.95,316.94) | 127.3(102.11,158.01) | 63.02(47.33,86.55) | 26.87(20.7,34.58) |

ASIR: age-standardized incidence rates; ASMR: age-standardized mortality rates; DALYs: disability-adjusted life years; ASDALYR: age-standardized disability-adjusted life years rates; UI: uncertainty interval. Note: Data are presented as estimate (95% UI) unless otherwise stated.

|  | 1990 |  |  | 2021 |  |  |
| --- | --- | --- | --- | --- | --- | --- |
|  | <20 years | 20-55 years | 55+  years | <20 years | 20-55  years | 55+  years |
| Incidence  number(95% UI) | 37801(22638,55329) | 340642(253604,428069) | 329118 (249073,429747) | 11423(6992,16537) | 244180(185466,305603) | 385505(313243,468258) |
| ASIR  (95% UI) | 8.49(5.09,12.43) | 57.94(43.14,72.81) | 229.32(173.55,299.44) | 3.42(2.09,4.95) | 34.42(26.14,43.07) | 101.73(82.66,123.57) |
| Death  number(95% UI) | 925(713,1185) | 13001 (10724,15535) | 43081 (35544,51660) | 54 (42,74) | 4028 (3002,5590) | 35416 (27790,45211) |
| ASMR(95% UI) | 0.21(0.16,0.27) | 2.21(1.82,2.64) | 30.02(24.77,36) | 0.02(0.01,0.02) | 0.57(0.42,0.79) | 9.35(7.33,11.93) |
| DALY  number(95% UI) | 76615 (59497,98302) | 651033 (540733,769869) | 952274 (790437,1133204) | 5153 (4034,6590) | 206175(158197,274856.) | 649356(516831,829613) |
| ASDALYR(95% UI) | 17.22(13.37,22.09) | 110.74(91.98,130.95) | 663.52(550.76,789.59) | 1.54(1.21,1.97) | 29.06(22.3,38.74) | 171.35(136.38,218.92) |

**Supplementary Table 2.** The number and age-standardized rates of incidence, deaths, and DALYs for PUD in China by age group, 1990 and 2021.

ASIR: age-standardized incidence rates; ASMR: age-standardized mortality rates; DALYs: disability-adjusted life years; ASDALYR: age-standardized disability-adjusted life years rates; UI: uncertainty interval. Note: Data are presented as estimate (95% UI) unless otherwise stated.

**Supplementary Table 3.** Results of the decomposition analysis for the change in PUD burden in China, 1990–2021.

|  | Overall_Difference | Aging(%) | Population(%) | Epidemiological_Change(%) |
| --- | --- | --- | --- | --- |
| Incidence number | -64142.3 | 365988.2(-570.59%) | 155542.2(-242.50%) | -585673(913.08%) |
| Death number | -17365.6 | 49269.84(-283.72%) | 12599.22(-72.55%) | -79234.6(456.27%) |
| DALY number | -807275 | 962371.2(-119.21%) | 323217.3(-40.04%) | -2092864(259.25%) |
|  |  |  |  |  |

Note: Negative or >100% percentage contributions indicate components that acted in the opposite direction to the net change and therefore partially offset the overall decline.


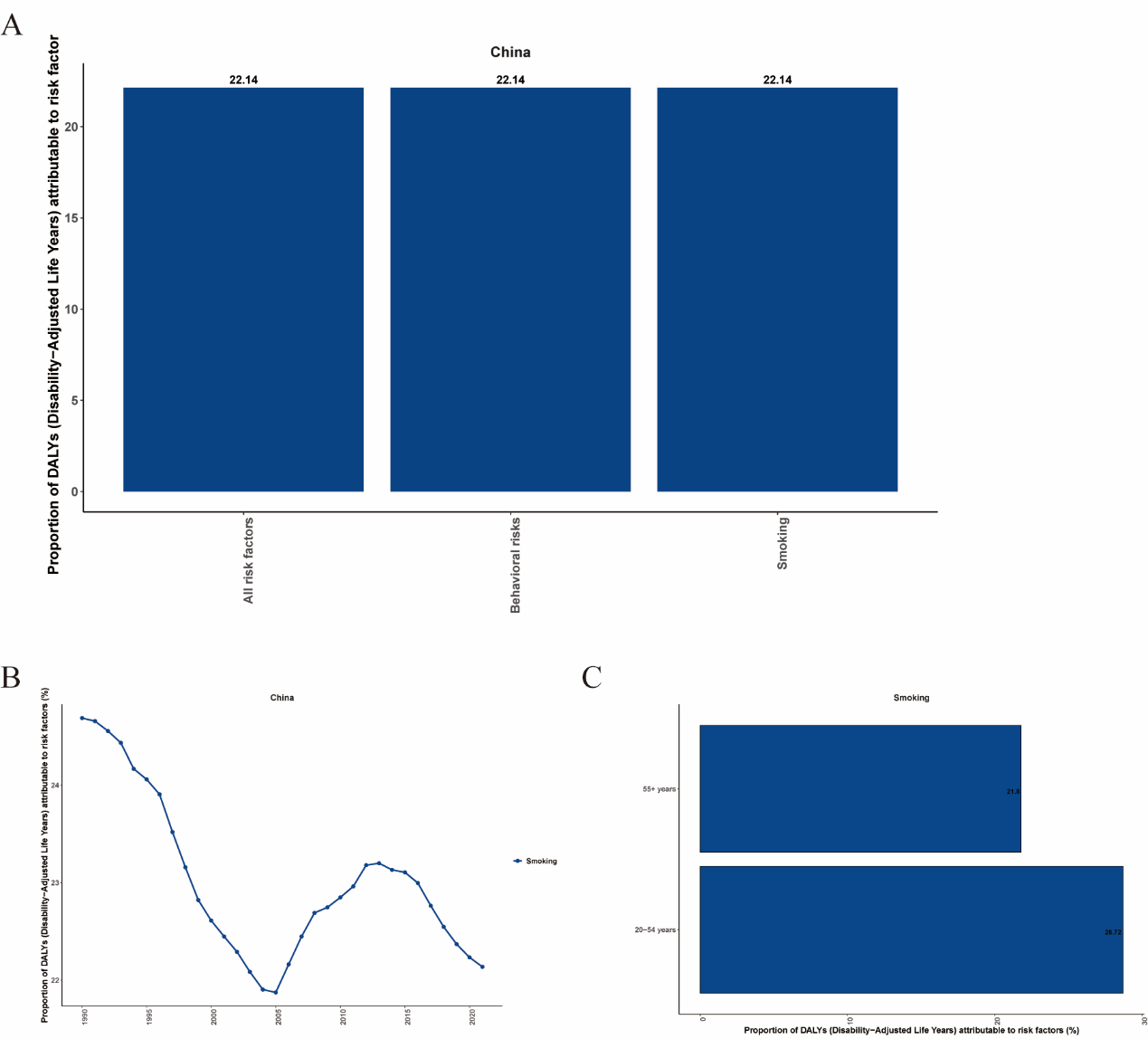


**Supplementary Figure 1. The proportion of PUD disability-adjusted life years (DALYs) attributable to risk factors in China, 2021.**

(A) The proportion of PUD DALYs attributable to smoking. (B) The temporal trend of the proportion of PUD DALYs attributable to smoking from 1990 to 2021. (C) The proportion of PUD DALYs attributable to smoking，stratified by age group.
